# RNA-binding proteins and their role in the regulation of gene expression in *Trypanosoma cruzi* and *Saccharomyces cerevisiae*

**DOI:** 10.1590/1678-4685-GMB-2016-0258

**Published:** 2017

**Authors:** Camila Oliveira, Helisson Faoro, Lysangela Ronalte Alves, Samuel Goldenberg

**Affiliations:** 1 Instituto Carlos Chagas, Fiocruz-Paraná, Curitiba, PR, Brazil.

**Keywords:** RNA-binding proteins, Trypanosoma cruzi, Saccharomyces cerevisiae, gene expression regulation

## Abstract

RNA-binding proteins (RBPs) have important functions in the regulation of gene expression. RBPs play key roles in post-transcriptional processes in all eukaryotes, such as splicing regulation, mRNA transport and modulation of mRNA translation and decay. RBPs assemble into different mRNA-protein complexes, which form messenger ribonucleoprotein complexes (mRNPs). Gene expression regulation in trypanosomatids occurs mainly at the post-transcriptional level and RBPs play a key role in all processes. However, the functional characterization of RBPs in *Trypanosoma cruzi* has been impaired due to the lack of reliable reverse genetic manipulation tools. The comparison of RBPs from *Saccharomyces cerevisiae* and *T. cruzi* might allow inferring on the function of these proteins based on the information available for the orthologous RNA-binding proteins from the *S. cerevisiae* model organism. In this review, we discuss the role of some RBPs from *T. cruzi* and their homologues in regulating gene expression in yeast.

## Introduction

Gene expression involves several events that occur at the transcriptional and post-transcriptional levels. The transcriptional control of gene expression has been extensively influenced by early work on bacterial transcription. However, in recent years, post-transcriptional events have gained much more attention. The pre-RNA undergoes extensive processing before the mRNA reaches its final destination and RNA-binding proteins (RBPs) associated to the RNA during its life-time play a key role in determining its fate in the cell. (Kishore *et al.*, 2010). The association of proteins with mRNAs is very dynamic and prone to changes according to the environment. Consequently RBPs are involved in the stabilization or destabilization of mRNAs in response to stress or extracellular signals (Alves and Goldenberg, 2016).

The availability of high-throughput analysis techniques, such as proteomics, has enabled the characterization of several RBPs. Nevertheless, the RBP network assembly and the mechanism of the RNA regulon are still poorly explored, and further work is required to determine the identity of all of the proteins and their respective roles in post-transcriptional events (Lunde *et al.*, 2007).

RBPs have one or multiple RNA-binding protein domains. The following are the best characterized RNA-binding domains: RNA Recognition Motif (RRM), K-homology domain (KH), RGG (Arg-Gly-Gly) box, zinc finger, double stranded RNA-binding domain (dsRBD), Pumilio/PUF domain and Piwi/Argonaute/Zwille (PAZ) domain (Finn *et al.*, 2010).

The RRM is the most abundant domain and also the most studied in RBPs (Afroz *et al.*, 2015). The information obtained from genome sequencing studies shows that RRM-containing proteins are present in all forms of life (Mari *et al.*, 2005). RRMs typically comprise approximately 90 amino acids and consist of four antiparallel β-strands (eventually they can have one or two short additional strands), which form a β-sheet that is packed against two α-helices, adopting the typical β1α1β2β3α2β4 conformation. The β3 and β1 strands of the RRM contain the RNP1 and RNP2 signature sequences, respectively (Cléry and Allain, 2012). Additionally, two or more RRMs can be combined in the same molecule to recognize longer stretches of RNA, with increased sequence affinity and specificity (Cléry and Allain, 2012).

The hnRNP K-homology (KH) domain comprises three α-helices around the surface of a central antiparallel β-sheet. Eukaryotic type I and prokaryotic type II KH domains share a minimal βααβ core, with two additional α and β elements positioned either in C-terminal (type I, eukaryotes) or N-terminal (type II, prokaryotes) orientation to this core motif (Grishin, 2001). This structure directs four nucleic acid bases towards a groove inside the protein structure where hydrophobic interactions and a network of main chain and side chain hydrogen bonds mediate nucleobase recognition. So far, protein domains with a classical KH fold but lacking a conserved GxxG motif have shown no nucleic acid-binding activity, although they interact with other nucleic acid binding domains and can modulate their RNA binding activity (Valverde *et al.*, 2008).

The RGG motif is an evolutionarily conserved sequence. In addition to the arginine and glycine repeats, aromatic residues are frequently observed in-between these sequences, and these residues may contribute to hydrophobic stacking within RNA bases. RGG/RG motives include RGG and RG repeats of varied lengths interspersed with spacers of different amino acids (Corley and Gready, 2008), and predicting the spacing that defines a functional RGG/RG motive is difficult. The structure of the RGG/RG has not been clearly defined due to its low sequence complexity.

Classical C2H2 ‘zinc finger’ proteins were identified as modular nucleic acid recognition elements, with two cysteine and two histidine residues that coordinate a zinc ion. Although mostly noted for their role as DNA-binding transcription factors, C2H2 zinc fingers were identified in the transcription factor IIIA (TFIIIA) (Vincent, 1986). TFIIIA contains nine C2H2 zinc fingers, which are used to recognize RNA and DNA targets. The zinc finger folds into a small domain comprising two β strands followed by one α helix. More recently, the C2H2 class of zinc finger protein has been shown to bind preferentially to RNA targets. These zinc fingers are characterized by three cysteine residues and one histidine residue that coordinate the zinc ion and form the Cys-X7-8-Cys-X5-Cys-X3-His sequence (Hall, 2005).

The dsRBD is a conserved protein domain of approximately 65–70 amino acids which binds to double-stranded or highly structured RNAs (Finn *et al.*, 2010). The dsRBD was first recognized as a conserved protein domain based on the similarities between *Drosophila* Staufen, human TAR-RNA binding protein (TRBP) and *Xenopus laevis* RNA-binding protein A (XlrbpA). The central function of dsRBDs is to bind to dsRNA regions, which is primarily achieved by recognizing specific RNA shapes. In addition to this major function, dsRBDs with protein-protein interaction properties have been reported to participate in the regulation of protein subcellular localization, suggesting that the participation of dsRBDs in nucleocytoplasmic trafficking is likely to represent a widespread auxiliary function of this type of RNA-binding domain (Banerjee and Barraud, 2014).

Pumilio is a family of sequence-specific RNA-binding proteins that regulate translation of the mRNA targets and also appear to interact with mRNA regulatory systems (Edwards, 2015). RNA recognition by Pumilio occurs through the PUF domain, named after its members Pumilio and FBF. Full-length Pumilio is a relatively large protein (156 kDa in *Drosophila*); however, only a fraction of the Pumilio protein (a 37 kDa fragment close to the protein C-terminus) is required for RNA binding, translational repression, and recruitment of other proteins. The PUF domain contains multiple tandem repeats of 35–39 amino acids which recognize specific RNA bases (Abbasi *et al.*, 2011).

The PAZ domain is found in Dicer and Argonaute proteins, two protein families with key roles in RNAi mechanisms. The PAZ domain consists of two subdomains, one of which displays OB-like folding (oligonucleotide/oligosaccharide binding). Hence, the PAZ motif might bind to single-stranded nucleic acids (Yan *et al.*, 2003). Crystallographic studies combined with biochemical approaches showed that the PAZ domain binds to ssRNAs with low affinity in a sequence-independent manner. A remarkable feature of the PAZ domain is that it can recognize the 3’-ends of ssRNAs. Both miRNAs and distinct types of small interfering RNAs (siRNA) are processed by the sequential action of RNase III enzymes (Drosha and Dicer in mammals, or Dicer alone in yeast and plants), which characteristically leave two 3’-overhangs on the processed products (Hutvagner and Simard, 2008).

## RNA-binding proteins in Trypanosomatids

The regulation of gene expression in trypanosomatids occurs mainly by post-transcriptional mechanisms. These protozoans present several peculiarities, such as a less condensed chromatin structure, polycistronic transcription, a *trans*-splicing mechanism, and the absence of canonical RNA polymerase II promoters. Genome analysis of the TriTryp database (containing genome sequences of the pathogenic *T. cruzi, Leishmania major* and *Trypanosoma brucei*) shows several RNA-binding proteins. Nonetheless, a comprehensive characterization of RNA-protein interactions remains elusive (Clayton and Shapira, 2007).

In 2005, De Gaudenzi and co-workers described approximately 80 proteins with RRM domains in *T. cruzi*, but few were functionally characterized (Table 1) (De Gaudenzi *et al.*, 2005). Another comprehensive study was conducted to characterize ribonucleoprotein complexes (mRNPs) in *T. cruzi* (Alves *et al.*, 2010). In this study, several RBPs were identified by proteomics, using polysomal and polysome-free fractions of exponentially growing epimastigotes and epimastigotes under conditions of nutritional stress.

**Table 1:** RNA binding proteins characterized in *Trypanosoma cruzi*.

Protein	Function	Ref.	Domain
SR62	mRNA processing/stability	Názer *et al.* (2011)	SR-related
ZC3H39	Regulator of a specific subset of mRNAs	Alves *et al.*, 2014	CCCH
UBP1	mRNA destabilizing factor	D’Orso and Frasch (2002)	RRM
UBP2	mRNA destabilizing factor	D’Orso and Frasch (2002)	RRM
PUF6	mRNA destabilizing factor	Dallagiovanna *et al.* (2008)	Pumilio
ZFP1	Involved in differentiation	Mörking *et al.* (2004)	CCCH
ZFP2	Involved in differentiation	Mörking *et al.* (2004, 2012)	CCCH
ZFP3	Involved in differentiation, translation regulator	Mörking *et al.* (2004)	CCCH
RBP40	Regulator of a specific subset of mRNAs	Guerra-Slompo *et al.* (2012)	RRM
RBP19	Involved in differentiation	Pérez-Díaz *et al.* (2012, 2013)	RRM
DRBD4/PTB2	Involved regulation of splicing	De Gaudenzi *et al.*, (2016)	RRM
PABP1	Involved in translation	Batista *et al.* (1994)	RRM

The life cycle of *T. cruzi* involves two hosts (triatomine insects and mammals) and comprises four morphological stages, two replicative (epimastigotes in the insects and amastigotes in the mammalian cells) and two infective forms (metacyclic trypomastigotes in the insects and bloodstream trypomastigotes in mammals). The epimastigotes differentiate in the midgut of the insect host and become metacyclic trypomastigotes, which are released in the excreta when the triatomine feeds on blood. The parasites penetrate the body of the mammalian host through the damaged skin or mucosa and invade different cell types. Within the cells, the parasites differentiate into amastigotes)De Souza, 2002).

## RNAi in *T. cruzi* and yeast

The canonical RNAi machinery comprises three main components: Dicer, Argonaute, and RNA-dependent RNA polymerase. Argonaute proteins contain two conserved domains, the PAZ and Piwi domains. These proteins are components of the RNA-induced silencing complex (RISC) (Liu *et al.*, 2004). Fungi, such as Ascomycetes, Basidiomycetyes, and Zygomycetes present the RNA silencing components in the genome, while few ascomycete and basidiomycete fungi apparently lost these components (Nakayashiki *et al.*, 2006).

*Saccharomyces cerevisiae, T.a cruzi, L. major* and *Plasmodium falciparum* do not have the RNAi machinery, which seems to have been lost or excessively simplified. However, an ORF encoding for an AGO/PIWI protein expressed in all stages of the life cycle of *T. cruzi* was recently described (Garcia-Silva *et al.*, 2010). The results showed that the TcPIWI-tryp is a canonical Argonaute in its domain architecture (Garcia-Silva *et al.*, 2010). Moreover, it was shown that the most represented sRNAs interacting with TcPIWI-tryp derived from rRNAs, which corresponded to known miRNAs of higher eukaryotes, indicating a possible evolutionary pathway of known canonical sncRNAs from structural RNAs (Garcia-Silva *et al.*, 2014).

## RBPs with RRM domain in *T. cruzi*

Some RBPs play an important role during the differentiation of the parasite by regulating the expression of specific transcripts. TcUBP-1 recognizes the AU-rich instability element located in the 3’-untranslated region (UTR) of mucin SMUG mRNAs (D’Orso and Frasch, 2002). TcUBP-2 binds to poly(U)-RNA and is differentially expressed during parasite development. Both proteins interact in the same complex and are implicated in controlling *T. cruzi* SMUG mucin mRNA levels. In addition, they are located preferentially in the polysomal fraction (D’Orso and Frasch, 2002).

TcRBP40 binds to AG-rich regions in the 3’-UTR of target mRNAs. Microarray data indicate that this protein binds to mRNAs encoding various transmembrane proteins. The TcRBP40 protein location varies throughout the parasite's life cycle. In the epimastigote stage It is localized in reservosomes, which are trypanosomatid organelles associated to protein and lipid storage, and in amastigotes and trypomastigotes it is dispersed in the cytoplasm, suggesting a potential gene regulatory function (Guerra-Slompo *et al.*, 2012).

TcRBP19 is differentially expressed during the life cycle of *T. cruzi* and is not detected only in the amastigote stage. Regulation of TcRBP19 is mediated by the 3’-UTR region, and the overexpression of TcRBP19 affects the *T. cruzi* life cycle and ability for infection (Pérez-Díaz *et al.*, 2012, 2013). Recently, De Gaudenzi *et al.* (2016), showed that TcDRBD4/PTB2 is an essential multifunctional RBP, involved in regulation of splicing, preventing trans-splicing and decreasing both UBP1 and UBP2 proteins expression.

TcPABP1 was first characterized in 1994 by Batista *et al.* (1994), showing that this protein has been conserved throughout eukaryotic evolution. This Poly (A) binding protein has been more extensively described in *T. brucei* than in *T. cruzi*. PABP1 and PABP2 are localized in different sets of granules in response to inhibition of either translation or *trans*-splicing. PABP2 co-localized with the marker DHH1 into RNP granules, which are similar to P-bodies, and in nuclear periphery granules, whereas PABP1 is localized in heat shock induced stress granules (Kramer *et al.*, 2013).

## RBPs with PUF domains in *T. cruzi*

The PUF family of RNA-binding proteins regulates their target mRNAs by binding to their 3’-UTR. In *T. cruzi*, the TcPUF6 protein is involved in the degradation of specific mRNAs, especially those that are upregulated in the infective trypomastigote form (Dallagiovanna *et al.*, 2008).

## RBPs with the CCCH zinc finger domain in *T. cruzi*

The *T. cruzi* proteins TcZFP1 and TcZFP2 have been characterized and contain the C2H2 domain. TcZFP1 binds specifically to oligoribonucleotides containing cytosine-rich sequences. This type of repetitive sequence is present in untranslated regions of many mRNAs in trypanosomatids (Mörking *et al.*, 2004). Ribonomic analysis showed that the targets of the protein TcZFP2 are associated with parasite-host interactions, for which expression is down-regulated in the replicative forms, indicating that TcZFP2 protein might act by destabilizing its targets (Mörking *et al.*, 2012). The protein TcZC3H39 sequesters highly expressed mRNAs and their associated ribosomes, slowing translation under stress conditions. In addition, the transcript content is changed in normal and stressful conditions, and most of its targets code for cytochrome c oxidase enzymes (COX) and ribosomal proteins, presenting evidence for the RNA regulon theory (Alves *et al.*, 2014).

## Other RBP domains in *T. cruzi*

Some RBPs involved in mRNA metabolism can be relocalized to the nucleolus in *T. cruzi* as a specific stress response. TcSR62 is an RBP that belongs to the SR-related protein family, which is implicated in several functions related to mRNA metabolism. TcSR62 is involved in mRNA processing/stability, since its overexpression in *T. brucei* affects the mRNA *trans*-splicing process and leads to a decreased abundance of several mRNAs (Názer *et al.*, 2011).

When mRNAs are not translated, they are compartmentalized into cytoplasmic structures named RNA granules. These RNA granules comprise the ‘processing bodies’ (‘P-bodies’) and the stress granules. Several RBPs have been implicated in the assembly and/or maintenance of these structures. TcDHH1, a putative DEAD-box RNA helicase, is involved in multiple RNA-related processes in various eukaryotes and accumulates in stress granules and P-bodies of yeast, animal cells and *T. brucei* (Kramer *et al.*, 2010). In *T. cruzi*, DHH1 is present in heavy protein complexes, which are not associated with the polysome complexes, and is located diffusely in the cytoplasm under normal conditions. However, DHH1 forms cytoplasmic granules upon nutritional stress or treatment with drugs that dissociate the polysomes (Holetz *et al.*, 2010).

## RNA-binding proteins in yeast

The RNA-RBP complexes can be identified by RBP immunoaffinity purification (RIP), where the proteins are purified together with the bound RNAs, and the associated RNAs can then be identified. CLIP (cross-linking and immuno-precipitation) is a method that can directly determine the binding sites of RBPs onto mRNA. A substantial number of mRNA-binding proteins from yeast were identified from studies on the mechanisms of biogenesis, localization, translation and degradation of mRNAs (Mitchell *et al.*, 2013).

## RBPs with an RRM domain in *S. cerevisiae*

**Figure 1:**
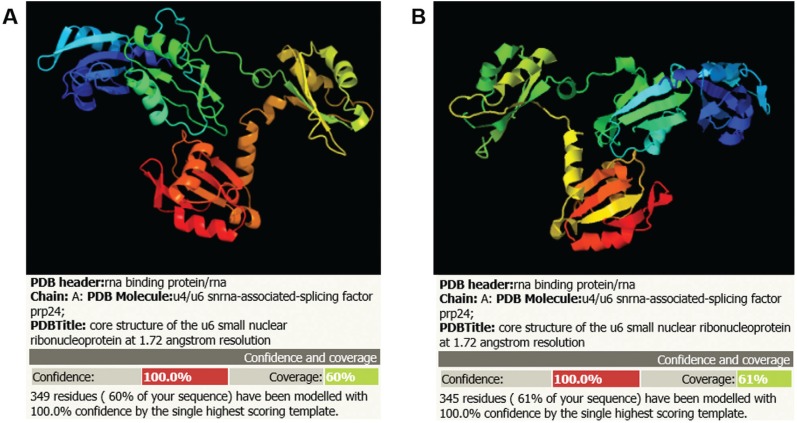
Structural prediction of ScPab1 (A) and TcPabp1 (B) proteins (Phyre2 program).

RBPs with RRM domains are well characterized in *S. cerevisiae*. This is the case of PABP1 (Poly-A binding protein), which contains four RRM domains (Figure 1), and is found in the cytoplasm, where it is associated with mRNA poly-A tails, stimulating translation initiation and regulating mRNA stability (Amrani *et al.*, 1997).

The second best studied protein in yeast is PUB1, which has three RRMs and can be located both in the nucleus and the cytoplasm, and is associated with poly(U) sequences (Anderson *et al.*, 1993). PUB1 is involved in the stabilization of mRNAs containing ARE (“AU-rich elements”), and it is also involved in the process of nonsense-mediated mRNA decay (NMD) (Ruiz-Echevarría and Peltz, 2000).

The ScPRP24 protein also contains three RRM domains and is involved in the formation and organization of the spliceosome complex (Shannon and Guthrie, 1991). Moreover, the RRM domains 2 and 3 of ScPRP24 stabilize the U6 RNA and allow it to complete the U4/U6 RNA interaction, thereby influencing the association and dissociation of U4 and U6 RNAs with ScPRP24 (Vidaver *et al.*, 1999).

## RBPs with PUF domain in *S. cerevisiae*

**Figure 2:**
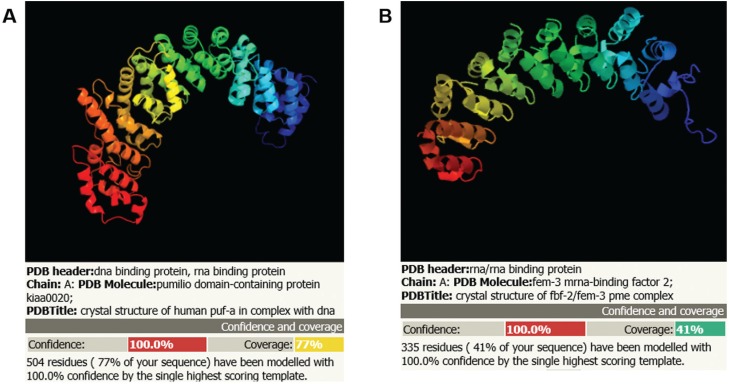
Structural prediction of ScPuf6 (A) and TcPuf6 (B) proteins (Phyre2 program).

Yeast possesses six PUF proteins (named PUF1–PUF6), and these proteins modulate mRNA stability through association with the 3’-UTR of their target mRNAs. For example, PUF1p activity involves recognition of UGUA sequences and surrounding sequences by PUF proteins. PUF also regulates several mitochondrial proteins, such as PMP1, PMP2, PMP3, and AST1. These mRNAs have been associated with PUF1p and/or PUF2p and encode membrane-associated proteins involved in proton transport (Ulbricht and Olivas, 2008). PUF3 promotes the deadenylation of Cox17 (Olivas and Parker, 2000), while PUF4 and PUF5 act on the deadenylation and decay of HO, a specific endonuclease that stimulates mating-type switching in budding yeast (Tadauchi *et al.*, 2001). Interestingly, PUF6 (Figure 2) acts on the regulation of Ash1, which represses HO in cells to block mating-type switching (Gu *et al.*, 2004).

## RBPs with zinc finger CCCH domains in *S. cerevisiae*

**Figure 3:**
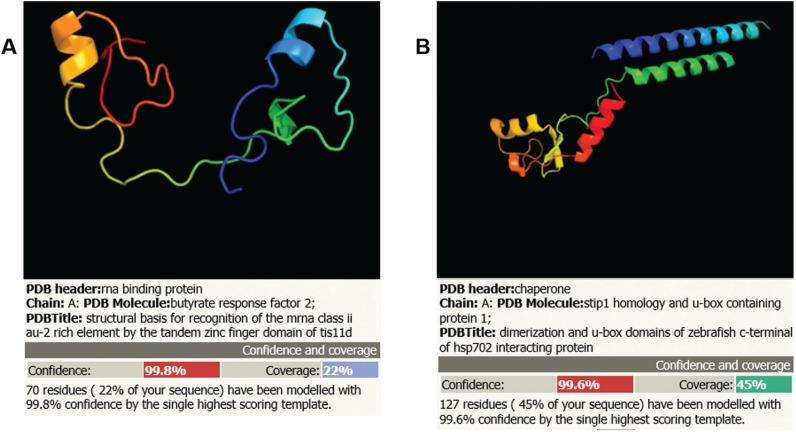
Structural prediction of ScCth1 (A) and TcZC3h39 (B) proteins (Phyre2 program).

CTH1 (Figure 3) and CTH2 were first described in yeast. Both proteins can play a role in mRNA activation or degradation of mRNA targets involved in iron homeostasis (Thompson *et al.*, 1996).

Two zinc finger proteins, MSN2 and MSN4, function as transcriptional activators (Estruch and Carlson 1993), and under stress conditions both proteins can activate one or more genes involved in the protective response following different types of stress (Martínez-Pastor *et al.*, 1996).

## Other RBP domains in *S. cerevisiae*

**Figure 4:**
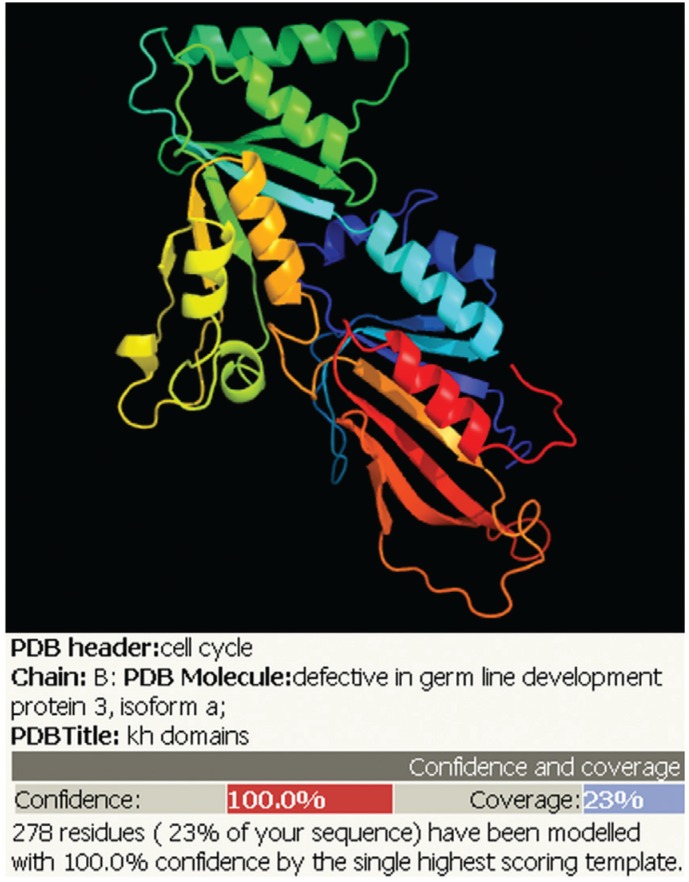
Structural prediction of ScScp160 protein (Phyre2 program).

There are many other RBPs that have been characterized. For example, SCP160 is a protein that has 14 repeats of the KH domain (Figure 4) and is associated with polyribosome bound mRNPs (Lang and Fridovich-Keil, 2000). Interestingly, this protein also participates in the formation of P-bodies, since it appears to prevent P-bodies formation under normal conditions (Weidner *et al.*, 2014).

## RBPs orthology between *T. cruzi* and *S. cerevisiae*

To investigate if the RBP proteins of *T. cruzi* are present in *S. cerevisiae* we performed an orthology analysis. The RBP amino acid sequences from *T. brucei* (De Gaudenzi *et al.*, 2005) were used to identify RBPs in *T. cruzi* through best reciprocal Blast hit analysis, resulting in 61 proteins with identity ranging from 87.04 to 30.38%. The identified proteins were then compared to all encoded proteins of *S. cerevisiae* genome using the same approach. A total of 20 *T. cruzi* proteins were found orthologous in *S. cerevisiae*, but the overall identity was lower, ranging from 44.44 to 22.17% (Table 2). Despite the low identity between *T. cruzi* and *S. cerevisiae* proteins, domain analysis showed that the proteins had related RBP domains, suggesting that these proteins are indeed orthologous between these two organisms.

**Table 2:** Orthology analysis of RNA-binding proteins between *T. cruzi* and *S. cerevisiae*.

*Trypanosoma cruzi* id (Tc)	Domains	*Saccharomyces cerevisiae* id (Sc)	Domains	Tc length	Sc length	Tc qcovhsp	Sc qcovhsp	pident
TcCLB.506885.70	RRM	sp|P04147|PABP	RRM	570	577	94	92	38.52
TcCLB.511741.40	RRM	sp|P40561|SGN1	RRM	231	250	39	35	43.33
TcCLB.504431.90	RRM	sp|P40567|MSL1	RRM	114	111	76	76	32.58
TcCLB.503577.20	Zinc Finger	sp|Q06102|YTH1	Zinc Finger	233	208	11	12	44
TcCLB.504071.80	RRM	sp|Q08920|NCBP2	RRM	188	208	60	68	40.85
TcCLB.511303.60	Eukaryotic translation initiation factor eIF2A / RRM	sp|P06103|EIF3B	Eukaryotic translation initiation factor eIF2A /RRM	696	763	90	83	22.17
TcCLB.511367.60	La / RRM	sp|P33399|LHP1	La / RRM	333	275	56	73	33.18
TcCLB.508299.89	RRM	sp|P53743|ESF2	RRM	238	316	44	34	32.41
TcCLB.511863.20	RRM	sp|P32605|RU1A	RRM	371	298	27	33	27.72
TcCLB.507037.20	RRM	sp|P37838|NOP4	RRM	486	685	70	53	27.59
TcCLB.507515.60	RRM	sp|P40565|IST3	RRM	156	148	40	43	44.44
TcCLB.504045.114	Tryptophan synthase alpha chain / RRM	sp|P00931|TRP	Tryptophan synthase alpha chain / RRM	185	707	54	14	25.23
TcCLB.510657.160	RRM	sp|Q08208|NOP12	RRM	421	459	50	48	24.68
TcCLB.503897.90	RRM	sp|Q06106|MRD1	RRM	878	887	99	77	27.14
TcCLB.504157.10	RRM	sp|P34167|IF4B	RRM	450	436	20	21	34.38
TcCLB.508409.80	MIF4G / RRM	sp|P39935|IF4F1	MIF4G / RRM	501	952	27	11	25.66
TcCLB.506693.30	RRM	sp|Q00539|NAM8	RRM	243	523	30	17	38.64
TcCLB.511127.10	RRM	sp|Q00916|RU17	RRM	240	300	33	26	33.75
TcCLB.508567.100	Adaptin N terminal region / RRM	sp|P38065|AP2A	Adaptin N terminal region / RRM	1405	1025	11	14	24.38
TcCLB.511867.180	PUB	sp|P32900|SKG6	Transmembrane alpha-helix domain	365	734	37	16	23.53

## Concluding remarks

RBPs are key players in gene expression regulation in all organisms. They allow the cells to change their expression profile very rapidly to respond to different types of stimuli. The fast response is particularly important in the case of unicellular organisms, such as trypanosomatids and yeast, that rapidly need to adapt to environmental changes to survive.

Despite the phylogenetic distance, in some cases, the function of a protein of interest is conserved. *S cerevisiae* is a powerful biological model because it is a simple eukaryote whose genome is easily manipulated and, therefore, can be used to obtain hints about the function of genes in another organism (Table 2). For example, the *T. cruzi* TcJ6 protein is a homologue of the Sis1 protein from *S. cerevisiae*, and these proteins are involved in translation initiation in both organisms (Salmon *et al.*, 2001). For instance, Mantilla *et al.* (2015) used *S. cerevisiae* to complement mutants for the *T. cruzi* protein TcP5CDH to study the proline metabolic pathway of the parasite.

The study of RBPs proteins and their function in unicellular eukaryotes should pave the way to enlighten the regulatory role of these proteins in higher eukaryotes.
